# Hsp90 Is Essential for Chl1-Mediated Chromosome Segregation and Sister Chromatid Cohesion

**DOI:** 10.1128/mSphere.00225-18

**Published:** 2018-06-06

**Authors:** Nidhi Khurana, Sayan Bakshi, Wahida Tabassum, Mrinal K. Bhattacharyya, Sunanda Bhattacharyya

**Affiliations:** a Department of Biotechnology and Bioinformatics, School of Life Sciences, University of Hyderabad, Hyderabad, India; b Department of Biochemistry, School of Life Sciences, University of Hyderabad, Hyderabad, India; University of Texas Health Science Center

**Keywords:** Chl1, Hsp90, chromosome segregation, sister chromatid cohesion

## Abstract

Recent studies have demonstrated that aberrant sister chromatid cohesion causes genomic instability and hence is responsible for the development of a tumor. The Chl1 (chromosome loss 1) protein (homolog of human ChlRl/DDX11 helicase) plays an essential role in the proper segregation of chromosomes during mitosis. The helicase activity of Chl1 is critical for sister chromatid cohesion. Our study demonstrates that Hsp90 interacts with Chl1 and is necessary for its stability. We observe that the Hsp90 nonfunctional condition (temperature-sensitive *iG170Dhsp82* strain at restrictive temperature) induces proteasomal degradation of Chl1. We have mapped the domains of Chl1 and identified that the presence of domains II, III, and IV is essential for efficient interaction with Hsp90. We have demonstrated that Hsp90 inhibitor 17-AAG (17-allylamino-geldenamycin) causes destabilization of Chl1 protein and enhances significant disruption of sister chromatid cohesion, which is comparable to that observed under the *Δchl1* condition. Our study also revealed that 17-AAG treatment causes an increased frequency of chromosome loss to a similar extent as that of the *Δchl1* cells. Hsp90 functional loss has been earlier linked to aneuploidy with very poor mechanistic insight. Our result identifies Chl1 as a novel client of Hsp90, which could be further explored to gain mechanistic insight into aneuploidy.

**IMPORTANCE** Recently, Hsp90 functional loss has been linked to aneuploidy; however, until now none of the components of sister chromatid cohesion (SCC) have been demonstrated as the putative clients of Hsp90. In this study, we have established that Chl1, the protein which is involved in maintaining sister chromatid cohesion as well as in preventing chromosome loss, is a direct client of Hsp90. Thus, with understanding of the molecular mechanism, how Hsp90 controls the cohesion machinery might reveal new insights which can be exploited further for attenuation of tumorigenesis.

## Abstract (precis)

Recently, Hsp90 functional loss has been linked to aneuploidy; however, until now none of the components of sister chromatid cohesion (SCC) have been demonstrated as the putative clients of Hsp90. In this study, we have established that Chl1, the protein which is involved in maintaining sister chromatid cohesion as well as in preventing chromosome loss, is a direct client of Hsp90. Thus, with understanding of the molecular mechanism, how Hsp90 controls the cohesion machinery might reveal new insights which can be exploited further for attenuation of tumorigenesis.

## INTRODUCTION

Genome stability is of paramount significance, and the failure to maintain it is associated with genetic diseases, abnormal immune responses, and susceptibility to cancer (1). The role of heat shock protein 90 (Hsp90) in maintaining genomic stability is well appreciated, as considerable progress has been made to understand the molecular mechanism that illustrates how Hsp90 inhibition synergizes with the radiation sensitivity of the cancer cell. Molecular insight into varied potential mechanisms through which Hsp90 orchestrates the DNA repair pathway unveils several major proteins of DNA break repair machinery as the clients of Hsp90 (2–5). In lower eukaryotes, it has been demonstrated that Hsp90 function is indispensable for homologous recombination (HR) and the stability of the Rad51 protein, which is a key player in searching for the homologous templates (6). Owing to the ability of Hsp90 to regulate gene expression, one of the previous works has established a link between Hsp90 abundance and genomic instability (7). Following various lines of evidence, Hsp90 has been shown to negatively regulate *RAD53* (DNA damage signaling kinase) transcription and thereby attenuate the DNA damage response pathway (7).

However, genome integrity is measured not only by the capability of the cells to repair broken DNA. A crucial factor which decides the fate of genome integrity is sister chromatid cohesion (SCC). A mutation in the family of genes that controls sister chromatid cohesion can cause various human diseases, and in all these cases, the patients suffer from increased genomic instability (8). Errors in the proper functioning of SCC lead to frequent improper segregation of chromosomes which eventually results in aneuploidy (9). Evidence suggests indirect implications of Hsp90 inhibition for sister chromatid cohesion. It has been found that the Hsp90-Sgt1 chaperone-cochaperone complex modulates the kinetochore assembly by providing stability to the Mis12 complex (10). However, Mis12 is a bona fide kinetochore protein and is not involved in sister chromatid cohesion. A recent study has shown that Hsp90 functional loss is linked to aneuploidy (11), but whether the effect of Hsp90 inhibition on chromosome segregation is because of instability of any component of the SCC machinery has not been studied until now.

A genome-wide screen by Zhao et al. in 2005 has revealed several interactors with Hsp90 (12); however, interaction with Chl1 could not be detected. Interestingly, in another screen with the N-terminal domain of Hsp90, Chl1 protein was found to interact. Earlier, it has been demonstrated that the *chl1* mutation induces mitotic chromosome loss and is responsible for increased frequency of spontaneous mitotic recombination (13). Chl1 has been found to function as an establishment factor in the cohesin complex responsible for efficient SCC (14). Its significant role in chromosome segregation is evident from the studies which show that loss of Chl1p leads to reduced retention of cohesin complex subunit (Scc1p) at centromeres, and *Δchl1* mutants lose sister centromere cohesion in both S phase and G_2_ phase (15). In mammals, ChlR1 is found to be crucial for embryonic development and in preventing aneuploidy, as it is required for binding of the cohesin complex to the centromere as well as the chromosome arms (16). ChlR1 in humans is unique in its ability to resolve the DNA triplex helix (17) and two-stranded antiparallel G quadruplex DNA (18) and thereby protects the cells from genomic instability. Biallelic mutations in the *CHLR1* gene in humans have been associated with the occurrence of the neurological disease termed Warsaw breakage syndrome (WABS) (19). Another family of WABS was identified with a mutation in the Fe-S domain (R263Q) of the *chlr1* gene which drastically reduces the DNA-dependent ATP hydrolysis activity of ChlR1, and hence, its helicase activity is significantly impaired (20). ATP binding mutants of both Chl1 (yeast) and ChlR1 (human) have been found to be compromised in their ability to perform the catalytic function in chromosome segregation (19–22). Interestingly, recent studies have claimed that under genotoxic stress conditions, the helicase activity of Chl1 is essential for its recruitment to the replisome but is dispensable for its function in SCC (23). A wide range of functions performed by this protein makes it an important target of study regarding its activity and regulation.

Sister chromatid cohesion is a crucial determinant of genome integrity as it facilitates the accurate flow of genetic material to daughter cells via faithful segregation of chromosomes and thereby provides the homologous template for DNA repair to occur. Among the cohesion establishment factors, Chl1 acts at the interface between DNA repair and sister chromatid cohesion. Its participation in DNA repair was reported in 2006 (24), where *Δchl1* mutants displayed hypersensitivity toward DNA-damaging agents. A study also suggests a more direct role of Chl1 and Ctf4 in homologous recombination (HR) repair, which is not directed via the establishment of SCC (25). ChlR1 depletion leads to the accumulation of DNA damage, and the defects are observed in the repair of DNA double-strand breaks during DNA replication (26).

Keeping in view the role of Hsp90 in the maintenance of genome integrity and the vital role of Chl1 and ChlR1 in efficient DNA repair, we intended to ask whether Hsp90 is essential for Chl1 stability and in mediating chromosome segregation and sister chromatid cohesion. To this end, we have used Saccharomyces cerevisiae as a model organism. We were able to show that Hsp90 and Chl1 physically interact, and using a yeast two-hybrid assay, we have determined the domains of Chl1 which are required for maintaining such interaction. Our study demonstrates that Hsp90 inhibition leads to the degradation of Chl1 and hence causes the abrogation of Chl1-dependent sister chromatid association and alters faithful segregation of chromosomes.

## RESULTS

### Inhibition of Hsp90 function causes destabilization of Chl1 and promotes its proteasomal degradation.

**FIG 1:**
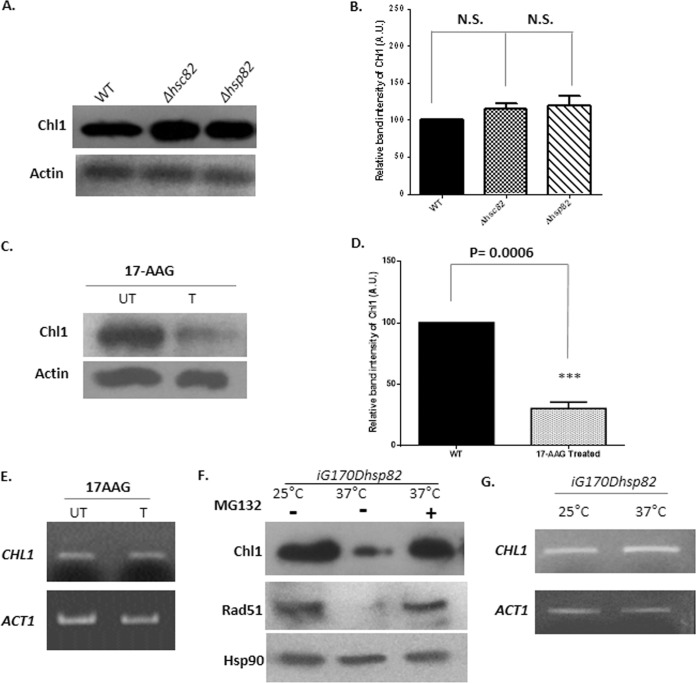
Inhibition of Hsp90 function causes destabilization of Chl1 and promotes its proteasomal degradation. (A) Western blot analysis shows the steady-state levels of Chl1 under single-knockout conditions of either form of yHsp90. Actin acts as a loading control. WT, wild type. (B) Quantification of the band intensities shows that Chl1 level remains unchanged under the conditions mentioned above. The band intensities in each lane are normalized against actin, and mean densities ± SDs are plotted. N.S., not significant. (C) Western blots revealing the effect of 17-AAG on the stability of Chl1 compared to the untreated condition. UT, untreated; T, treated. (D) Quantification of the band intensities from three different experiments displays 3-fold reductions in the level of Chl1 upon loss of function of Hsp90. The band intensities are normalized against actin, and mean densities ± SDs are plotted. (E) Semiquantitative reverse transcription-PCR analysis showing no effect on *CHL1* transcript levels under 17-AAG-treated condition normalized against *ACTIN*. (F) Western blot analysis showing the reaccumulation of Chl1 upon the inhibition of the proteasome degradation pathway when Hsp90 is nonfunctional. The experiment was carried out in a temperature-sensitive strain at permissive and restrictive temperatures. Levels of Hsp90 remain unaffected owing to its regulation under the noninducible *GPD* promoter. (G) Semiquantitative reverse transcription-PCR analysis showing no effect on *CHL1* transcript levels under permissive and restrictive temperatures normalized against *ACTIN*.

In order to understand the role of Hsp90 in Chl1-mediated proper segregation of chromosomes, we first set out to determine whether yeast Hsp90 (yHsp90) is required for Chl1 stability. Yeast has two paralogs of Hsp90: Hsp82, which is the inducible form, and Hsc82, which is the constitutively expressed form. Hence, to figure out the effect of yHsp90 on Chl1p, the steady-state level of Chl1p was studied under single-knockout conditions as *Δhsp82* and *Δhsc82* mutants. Western blot analysis was performed to investigate the steady-state level of Chl1 (Fig. 1A). The quantification of band intensities in Fig. 1B shows that under single-knockout conditions, i.e., *Δhsp82* and *Δhsc82* mutants, the levels of Chl1p remain unaffected. This observation can be attributed to the functional redundancy observed between the two isoforms of Hsc82 and Hsp82. To overcome the obstacle of functional redundancy of Hsp82 and Hsc82, we treated the cells with 40 µM 17-AAG for 18 h to inactivate both the isoforms. The Western blot analysis of 17-AAG-treated cells is compared with that of the untreated cells, and Fig. 1C shows that Chl1 levels are significantly reduced in treated cells. The quantification of band intensities in Fig. 1D reveals that the steady-state level of Chl1p is reduced by 3-fold under the condition where the yHsp90 function is lost. However, there is no reduction in the *CHL1* transcript under such a condition (Fig. 1E). It was earlier established that inhibition of Hsp90 induces proteasomal degradation of the proteins that are Hsp90 clients (27). So, we monitored Chl1 protein level in the presence and absence of proteasome degradation pathway inhibitor MG132 (Fig. 1F). For this experiment, we have used a temperature-sensitive strain, *iG170Dhsp82* (28), which is a double knockout for endogenous Hsc82 and Hsp82 and harbors a TS (temperature-sensitive) mutant of *hsp82* at the *HIS3* gene locus under the noninducible *GPD* (glyceraldehyde-3-phosphate dehydrogenase) promoter. This copy bears an alteration of glycine to aspartic acid at a conformationally restrictive position which leads to an improper folding of Hsp82 at a high temperature such as 37°C. Consequently, the function of Hsp82 is compromised at 37°C; however, at a lower temperature such as 25°C, the strain behaves as wild type, keeping the Hsp82 function intact. We incubated the strain at 37°C with a potent proteasome inhibitor, MG132, at the concentration of 50 µM. The protein extract was isolated from the cells growing under three conditions—at 25°C (wild-type condition), 37°C (Hsp90 loss-of-function condition), and 37°C with MG132 supplementation in the medium (proteasome inhibition along with Hsp90 functional loss). Hsp90 level was used as a loading control, because in this strain Hsp90 is expressed from a plasmid under the control of the constitutively expressed GPD promoter. Thus, its level should not change at a different temperature or with MG132 treatment. Upon analyzing the Western blot, we have found that MG132 treatment resulted in the accumulation of Chl1, which supports the idea that the proteasome pathway is involved in regulating basal levels of Chl1 (Fig. 1F). To confirm the efficiency of treatment by MG132, Rad51 (client of Hsp90) levels were monitored under the same conditions, and we observed that Rad51 levels are also accumulated upon MG132 treatment, which accords with our previous finding (6). Taken together, these data suggest that Hsp90 chaperone activity is required to maintain the endogenous level of Chl1 and that inhibition of Hsp90 function induces the proteasomal degradation of Chl1. We did not, however, observe any reduction in the *CHL1* transcript in the *iG170Dhsp82* strain while it was grown at the restrictive and permissive temperature (Fig. 1G). Thus, it suggests that destabilization of Chl1 is likely the major mechanism for Chl1 loss under the Hsp90-inactivated condition.

### Hsp90 physically interacts with Chl1.

**FIG 2:**
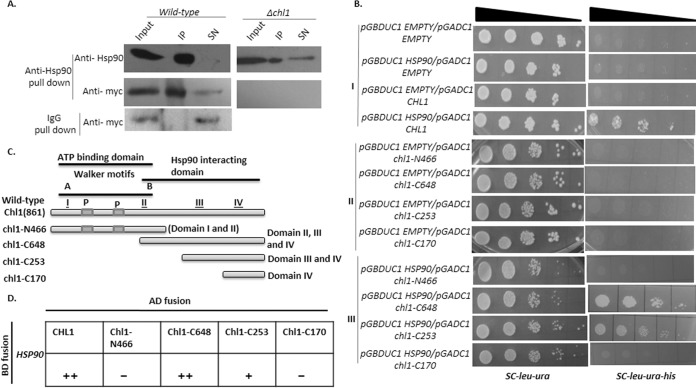
Hsp90 physically interacts with Chl1. (A) Coimmunoprecipitation data showing the interaction of Hsp90 with Chl1. The pulldown assay was performed with anti-Hsp90 antibody as well as with preimmune sera, and the assay mixture was immunoblotted with anti-Myc antibody to probe Chl1. IP, immunoprecipitate; SN, supernatant. (B) The right panels show yeast two-hybrid analysis exhibiting the extent of the interaction between Hsp90 and different constructs of Chl1. To study the protein-protein interaction, equal numbers of cells were serially diluted and spotted on medium lacking uracil, leucine, and histidine. The left panels show various combinations of bait and prey vectors. They show that all the strains are viable under normal conditions. In the right panels, growth is displayed only by the strains harboring Hsp90-bait vector and Chl1-prey vector, which indicates that interaction exists between these proteins. (C) Schematic representation of the domain organization of full-length Chl1 and different truncated versions created for interaction analysis with Hsp90. P, PEST sequences. (D) Interaction evaluated according to growth in triple dropout medium: strong (++), weak (+), or no (−) interaction.

In view of being a client of Hsp90, the protein must physically interact with Hsp90. To study whether Chl1 exhibits physical interaction with Hsp90, we performed a coimmunoprecipitation (Co-IP) assay. The result demonstrated that a significant amount of Chl1 protein is associated with Hsp90. However, the control strain lacking Chl1 showed no detectable background, although a significant proportion of Hsp90 was immunoprecipitated from cellular extract (Fig. 2A). The interaction between Hsp90 and Chl1 was further validated using yeast two-hybrid (Y2H) analysis. Full-length *CHL1* was cloned into a prey vector (p*GADC1*), and full-length *HSP90* was cloned in a bait vector (p*GBDUC1*). After transformation of these plasmids into the PJ69-4A strain, the transformants were scored for interaction on synthetic medium plates lacking histidine (for *HIS3* reporter gene activity). The results indicate that Chl1 interacts with Hsp90 (Fig. 2B, panel I). To map the domains of Chl1 which are crucial for interaction with Hsp90, we cloned four truncated regions of *CHL1* in the p*GADC1* vector. The schematic representation is given in Fig. 2C. The first construct comprises the N-terminal 466 amino acids of Chl1 (N-466) (29). This will express domain I (Walker A motif) and domain II (Walker B motif) of the Chl1 protein, which contain its ATP binding domain. Between domain I and domain II, there are two PEST sequences (P1 and P2), the presence of which reduces the stability of a protein. The second construct comprises 648 amino acids of Chl1 (C-648) that express domains II, III, and IV of Chl1 protein. Thus, it lacks the Walker A motif along with two PEST sequences. The third construct contains domain III and domain IV of Chl1. The fourth construct comprises the last 160 amino acids containing the C-terminal domain, which expresses domain IV. We transformed four different constructs of *chl1*-fused prey vectors to PJ69-4A cells harboring p*GBDUC1/HSP90*, and they were plated in synthetic medium lacking histidine (Fig. 2B, panel III). We observed substantial interaction when domains II, III, and IV were present. We also found feeble interaction when domains III and IV were present. However, the presence of domains I and II together was not sufficient to ensure interaction. Similarly, domain IV alone did not show any interaction. The extent of interaction between various truncated forms of Chl1 and Hsp90 is schematically represented (Fig. 2C). From our experiments, it can be concluded that there are several contact points between Chl1 and Hsp90 which span domains II, III, and IV of Chl1. Deletion of any part of this region eliminates the strong interaction between Chl1 and Hsp90.

### Inhibition of Hsp90 function exhibits a defect in chromosome segregation to the same extent as that of the *chl1* deletion mutant.

**FIG 3:**
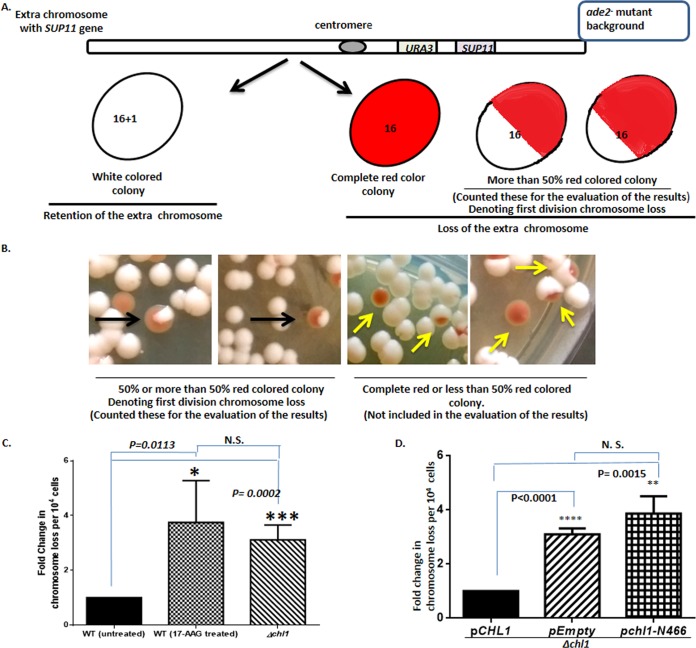
Inhibition of Hsp90 function exhibits a defect in chromosome segregation to the same extent as that of the *chl1* deletion mutant. (A) Schematic representation of the chromosome loss assay, indicating the possible outcome of different-color colonies. (B) Representative images of the colonies obtained. The black arrows indicate the colony with first-division chromosome loss; the yellow arrows show the colonies that are not considered for the analysis, as a fully red colony denotes chromosome loss before plating and a colony less than 50% red denotes chromosome loss later than the first division. (C) Bar graph showing fold change in chromosome loss frequency exhibited by the wild-type strain, Hsp90-inhibited strain, and *Δchl1* strain. (D) Bar graph showing fold changes in chromosome loss frequency exhibited by the null *chl1* strain harboring the truncated Chl1 (N-466) that blocks the interaction with Hsp90. The experiment was done in the presence of an isogenic positive control (null *chl1* strain harboring full-length *CHL1*) and a negative control (the same strain harboring empty plasmid). The number of colonies showing first-division chromosome loss for each condition was obtained from three different sets of experiments. Error bars indicate SDs (*n* = 3). *P* values were calculated using the two-tailed Student *t* test; NS, not significant.

Chl1 prevents chromosome loss from the cells, and Hsp90 has been associated with the chromosome loss phenotype earlier (11). However, we wanted to determine whether the extent of chromosome loss under the 17-AAG treatment condition is similar to what would occur under the *Δchl1* condition. To that end, we used an assay strain where one nonessential chromosome was inserted which carries a *SUP11* gene that suppresses *ade2* mutation of the assay strain (30). Retention of the extra chromosome (the 17th chromosome) allows the cell to bypass the *ade2* null phenotype, and the cells grow as a white colony. However, the loss of the extra chromosome (the total number of chromosomes is 16) produces a red-pigmented phenotype like that of the *Δade2* mutant, implying loss of *SUP11*. This strain was made *Δchl1* to score for the frequency of chromosome loss under such conditions. The *PDR5* gene was knocked out to ensure the uptake of 17-AAG. The frequency of first-division chromosome loss was evaluated for this experiment. Figure 3A shows the schematic representation of chromosome loss assay with the possible outcome of the different-color colonies. Figure 3B represents the images of the kinds of colonies obtained under different conditions, i.e., wild-type cells, 17-AAG-treated cells, and *Δchl1* cells. The first two panels show more than or at least 50% red colonies, which implies first-division chromosome loss, and these were the kinds of colonies taken into account. The third and fourth images correspond to the colonies which were excluded from the analysis as they were either a completely red colony or less than 50% red sectored, implying that the chromosome loss happened either before plating or later than first division. Upon analysis, we found that the frequency of first-division chromosome loss obtained for the *Δchl1* and Hsp90 inhibition conditions was 4- and 5-fold higher than the wild type, respectively, but similar to each other (Fig. 3C). Next, we wanted to determine whether the interaction between Chl1 and Hsp90 is crucial for the chromosome segregation function of Chl1. Earlier, it had been demonstrated that the ATP binding site of Chl1 is essential for chromosome segregation (31). However, it is not known whether other domains of Chl1 are required for chromosome segregation. To decipher that, we expressed truncated Chl1 (domain I and II) in the *Δchl1* strain and performed a chromosome loss assay. We generated isogenic positive and negative controls by expressing full-length Chl1 and empty vector in the *Δchl1* strain. Our result shows that the absence of the Hsp90-interacting region of Chl1 causes a 4-fold increase in chromosome loss compared to the full-length Chl1 (Fig. 3D). Thus, the presence of the ATPase domain (domains I and II) alone is not sufficient to prevent chromosome loss. The presence of the entire Hsp90-interacting domain (domains II to IV) is also required.

### Inhibition of Hsp90 is associated with a reduction in sister chromatid cohesion.

**FIG 4:**
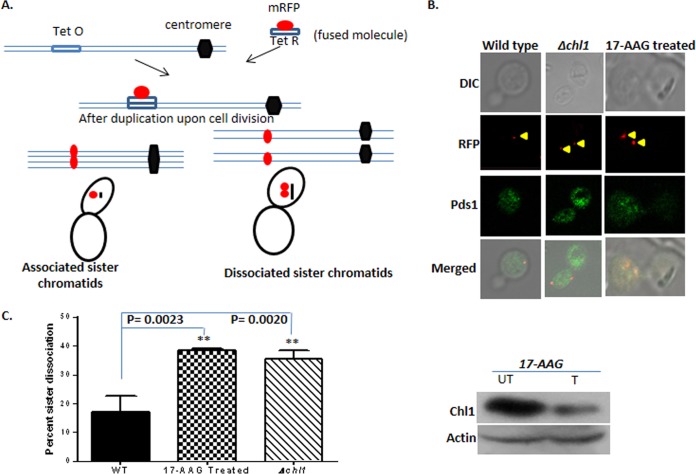
Inhibition of Hsp90 is associated with a reduction in sister chromatid cohesion. (A) Illustration of the principle behind the assay. The mRFP (monomeric red fluorescent protein) molecule fused with the Tet repressor indirectly labels the chromosome, which can be visualized under the confocal microscope. The cells are arrested with nocodazole at preanaphase. After duplication of chromosomes, if the sister chromatids are associated, then that will appear as one red focus, and if they are dissociated, then that will appear as either two distinct foci or one diffused focus. (B) Representative images for different strains (wild type, *Δchl1*, and 17-AAG treated) are shown. The top panel shows the cells in bright field. The second panel depicts the associated/dissociated sister chromatids marked by red foci which are indicated by yellow arrowheads in a single cell. The third panel shows the expression of Pds1p as marked by green fluorescence. The bottom panel shows the merged image. DIC, differential interference contrast. (C) Graph showing average percentage of cells displaying dissociated sister chromatids from three different sets of experiments. For each strain, at least 1,000 cells were counted. The error bars represent standard deviations. The Western blot panel at right confirms that Chl1 protein levels are diminished upon Hsp90 inhibition by 17-AAG treatment. UT, untreated; T, treated.

Since the stability of Chl1 depends upon Hsp90, we wanted to investigate whether the loss-of-function mutation of Hsp90 would affect Chl1 function in sister chromatid cohesion. Chl1 is known to promote the loading of Scc2 and cohesion proteins on DNA and thereby plays a critical role in sister chromatid cohesion (32). The strain lacking *chl1* exhibits severe chromosome segregation and cohesion defects (13, 14). We intended to assess sister chromatid dissociation upon Hsp90 inhibition. For this purpose, we used a strain, NKY4, where *TET* operator sites are integrated at the *URA3* location at chromosome V. Expression of red fluorescent protein (RFP)-tagged Tet repressor protein (RFP-TetR) allows the visualization of the sister chromatids. The illustration of the principle behind this assay is shown in Fig. 4A. We have tagged the Myc epitope at the chromosomal locus of *PDS1* so that we can use indirect immunofluorescence to visualize Pds1 expression as a marker for preanaphase cells. We knocked out *chl1* from the assay strain, which was used as the negative control in our assay. To determine the effect of Hsp90 inhibition on sister chromatid cohesion, we treated the assay strain with 17-AAG. In order to achieve maximum uptake of 17-AAG, *PDR5* was knocked out in the assay strain. To assay for the cohesion defect, we used the nocodazole-arrested wild-type strain, the 17-AAG (Hsp90 inhibitor)-treated strain, and the *Δchl1* strain. To verify that cells were arrested at the preanaphase stage, we monitored cell morphology and Pds1 expression. Pds1-positive cells were monitored for RFP-tagged chromosomal loci. The wild-type cells predominantly produced single red dots, indicating that sister chromatids are tightly associated. On the other hand, *Δchl1* cells mostly showed two closely spaced dots, indicating that the sister chromatids are dissociated (Fig. 4B). Our study showed that in wild-type cells only 17% of sister chromatids are dissociated, whereas the *Δchl1* strain showed 35% sister chromatid dissociation. These results strongly corroborate previous reports (14), suggesting that the assay system is reproducible. Once the assay was established, we wanted to investigate the effect of Hsp90 inhibition on sister chromatid cohesion. We observed that 17-AAG treatment causes a significant increase in the amount of sister chromatid dissociation (about 38%) (Fig. 4C), which is comparable to that found in the case of *Δchl1* strains. The accompanying Western blot in this figure confirms that Chl1 is significantly destabilized upon 17-AAG treatment. These data put forward the critical role of Hsp90 in preventing missegregation of chromatids among daughter cells under normal conditions.

## DISCUSSION

In recent years, several studies have established the important role of Hsp90 in maintaining genome integrity. Multiple components of DNA double-strand break repair pathways have been demonstrated to be the direct client of Hsp90 (5). In this work, we have established for the first time that Chl1, one of the major components of sister chromatid cohesion machinery, serves as a bona fide client of Hsp90. Genome-wide yeast hybrid screens identified that the N-terminal domain of yHsp90 (1 to 220 amino acids) interacts with Chl1, but such studies failed to detect any interaction with the full-length Hsp90 (12). Our study demonstrates that the full-length Hsp90 could also associate itself with Chl1. We have also confirmed the physical association between Chl1 and Hsp90 using coimmunoprecipitation. Hsp90 shows a variable degree of association with its clients. Some of the clients like heat shock factor, steroid hormone receptors, Rad51, etc., remain associated with Hsp90 to maintain their functional form. However, Hsp90 kinase clients are primarily associated with Hsp90 through transient interaction, and once chaperoned, they are readily released from Hsp90 as a functional protein. As a result, they are not detectable in coimmunoprecipitation (33). In the case of Chl1, we found that a significant proportion of Chl1 remains associated with Hsp90 as detected in coimmunoprecipitation. This indicates that Chl1 requires continuous association with Hsp90 to main its stability.

The exquisite importance of Chl1 in cohesion establishment came into light when it was shown to play a role in the Eco1-independent cohesion stabilizing pathway. Chl1 and Ctf4 (cohesion establishment factors) contribute to cohesion establishment in a way distinct from the mere cohesin stabilization on the chromosomes (34). This study describes the importance of the contribution of Chl1 in sister chromatid cohesion. Our results for the first time demonstrate that the stability of Chl1 depends on Hsp90 and inhibition of Hsp90 leads to sister chromatid dissociation. We do not rule out the possibility that 17-AAG-mediated increased percent sister chromatid dissociation may occur due to the inactivation of additional clients of Hsp90 in the cohesion family. However, the fact that Hsp90 inhibition also results in a defect in chromosome segregation strongly suggests that Chl1 is one of the main clients of Hsp90 that controls aneuploidy, as the mutation of Chl1 has been shown to produce defects in both chromosome segregation and sister chromatid cohesion. To establish specificity toward Hsp90 inhibition and Chl1 stability, we tried to overexpress Chl1 under the Hsp90-inactivated condition and look for the reversal of phenotype. However, our work showed that the overexpression of Chl1 manifests a dominant negative effect (data not shown). It may be possible that a higher abundance of Chl1 may have detrimental effects on the cell. This may be explained by the presence of two PEST sequences at the amino-terminal domain of Chl1 which destabilize the protein. Our study also shows that the interaction between Hsp90 and Chl1 is essential for Chl1-dependent chromosome segregation. This conclusion is derived from our finding that in cells harboring truncated Chl1, where Hsp90-interacting domains are absent, the chromosome loss frequency is comparable to that of cells devoid of Chl1, and it is enhanced by 4-fold compared to the cells harboring wild-type Chl1. However, we cannot formally rule out the possibility that the truncated version of the Chl1 protein might lack other important but unidentified domains which might be required for Chl1-mediated chromosome segregation.

Earlier studies have shown that *CHL1*-deficient cells are more sensitive to DNA-damaging agents such as methyl methanesulfonate (MMS) and UV radiation than are the wild-type cells (35). Thus, any condition that destabilizes Chl1 should also reduce cell survivability under DNA-damaging conditions. Previously, we have also observed that the *HSP90*-inactivated condition causes sensitivity toward DNA-damaging agents in S. cerevisiae (6), and our work established that the important proteins of homologous recombination such as Rad51 and Rad52 are destabilized under such conditions. As the establishment of sister chromatid cohesion is essential for HR, destabilization of Chl1 may also be responsible for inefficient HR and decreased survivability under DNA-damaging conditions. Since Hsp90 may have several clients involved in the DNA repair pathway, we have monitored specialized functions of Chl1 under the Hsp90-inactivated condition. The increased frequency of chromosome loss, as well as increased frequency of sister chromatid dissociation, pinpoints that Chl1 function is lost specifically under the Hsp90-inhibitory condition.

The deregulated activity of Chl1/ChlR1 has been associated with cancer. The deregulated expression of ChlR1 leads to its amplification in a variety of tumors like melanomas, breast cancer, ovarian cancer, and pancreatic and lung cancer. Our study has important implications for cancer therapy as recent studies have shown that inhibition of sister chromatid cohesion along with anaphase-promoting complex (APC/c) leads to the fetal mitotic arrest in several cancer cell lines (36). Thus, understanding the molecular mechanism of how Hsp90 controls the cohesion machinery might reveal new target molecules within the cohesion family, which can be explored further to understand the molecular mechanism of formation/development of tumors in humans.

## MATERIALS AND METHODS

### Plasmids.

The plasmids used for yeast two-hybrid assays were p*GBDUC1* as the bait vector and p*GADC1* as the prey vector (37). We have amplified the full-length *HSP82* using the primer pairs OSB 21 and OSB 22 and cloned it into the bait vector. Similarly, the full-length *CHL1* and truncated versions of *chl1* were amplified using the primer pairs OSB 90-OSB 91, OSB 90-OSB 381 (which amplifies bp 1 to 1398 of *chl1*), OSB 382-OSB 91 (which amplifies bp 640 to 2586 of *chl1*), OSB 383-OSB 91 (which amplifies bp 1825 to 2586 of *chl1*), and OSB 384-OSB 91 (which amplifies bp 2074 to 2586 of *chl1*), generating full-length *CHL1*, *chl1-N466*, *chl1-C648*, *chl1-C253*, and *chl1-C170* (as presented in Fig. 2C), respectively, and cloned into the prey vector. Full-length *CHL1* and *chl1-N466* were subcloned into 2μ yeast expression vector pLA, which expresses *CHL1* under the control of the GPD promoter. The C-terminal *MYC* tagging of *CHL1* at the chromosomal locus was done by using pFA6a-13*MYC-KANMX* and pFA6a-13*MYC-TRP* vectors (38) as a template. The primers used for *MYC* tagging of *CHL1* and confirmation of the tag were OSB 78, OSB 79, and OSB 80, respectively. The knockout of *CHL1* was achieved by using pFA6a-TRP and pFA6a-HIS vectors as the templates and primers OSB 107 and OSB 108 (38). To confirm the generation of the *Δchl1* knockout strain, OSB 109 and OSB 108 were used. To knock out *PDR5*, the knockout cassette pFA6a-TRP (38) was amplified using the primer pairs OMKB 411 and OMKB 412. The *Δpdr5* knockout strain was confirmed using the primer pair OMB 413 and OSB 290. Another strategy taken up to knock out *PDR5* in NKY2 was the amplification of the knockout cassette at the *PDR5* locus from the SLY89 strain using the primers OSB 318 and OSB 319. For the amplification of *CHL1* cDNA, we have used the OSB 160 and OSB 91 primer pair, and for the amplification of *ACTIN* cDNA, the primer pair OSB 16 and OSB 14 was used. Sequences of all the primers used in this paper are shown in Table 1.

**TABLE 1:** Primers used in this study

Primer name	Sequence	Purpose
OSB 90	5′ CTG TGG ATC CAT GGA CAA AAA GGA ATA TTC 3′	Forward primer used to amplify full-length *CHL1*
OSB 91	5′ CGA TGT CGA CTT AGC GTG AAT TCA GGC TGC 3′	Reverse primer used to amplify full-length *CHL1*
OSB 78	5′ AAC ACG GAA GTT TTT TTC AAT GCG CAG CCT GAA TTC ACG CCG GAT CCC CGG GTT AAT TAA 3′	Forward primer used to generate *MYC* tag at the C-terminal end of *CHL1* at the chromosomal locus
OSB 79	5′ ATA TAG TAG TAA TCA CAG TAT ACA CGT AAA CGT ATT CCT TGA ATT CGA GCT CGT TTA AAC 3′	Reverse primer used to generate *MYC* tag at the C-terminal end of *CHL1* at the chromosomal locus
OSB 80	5′ CGG CAT GCA AAT GAT TAC GC 3′	Forward primer used to confirm *MYC* tagging of *CHL1*
OSB107	5′ GTA GAA AAC CAG GCT AAA AAC AGT CAC ACT AGT CCA AAA ACG GAT CCC CGG GTT AAT TAA 3′	Forward primer used for *CHL1* knockout
OSB 108	5′ ATA TAG TAG TAA TCA CAG TAT ACA CGT AAA CGT ATT CCT TGA ATT CGA GCT CGT TTA AAC 3′	Reverse primer used for *CHL1* knockout
OSB 109	5′ CGT AAC CAC AGA GTT GAG GTA G 3′	Forward primer used for *CHL1* knockout confirmation
OMKB 411	5′ AAG TTT TCG TAT CCG CTC GTT CGA AAG ACT TTA GAC AAA ACG GAT CCC CGG GTT AAT TAA 3′	Forward primer used for *PDR5* knockout using pFA6a-TRP plasmid
OMKB 412	5′ TCT TGG TAA GTT TCT TTT CTT AAC CAA ATT CAA AAT TCT AGA ATT CGA GCT CGT TTA AAC 3′	Reverse primer used for *PDR5* knockout using pFA6a-TRP plasmid
OMKB 413	5′ AAG TCA CGC AAA GTT GCA AAC 3′	Forward primer used for confirmation of *PDR5* knockout
OSB 290	5′ CCG TAA TCA TTG ACC AGA GCC 3′	Reverse primer used for confirmation of *PDR5* knockout using pFA6a-TRP plasmid
OSB 318	5′ CTG TTG AAC GTA ATC TGA GC 3′	Forward primer used for *PDR5* knockout from SLY89 strain
OSB 319	5′ TTC TCG GAA TTC TTT CGG AC 3′	Reverse primer used for *PDR5* knockout from SLY89 strain
OSB 160	5′ GGA AGA GGA AGC TTC ACG AG 3′	Forward primer used to amplify *CHL1* for semiquantitative PCR
OSB 91	5′ TTA GCG TGA ATT CAG GCT GC 3′	Reverse primer used to amplify *CHL1* for semiquantitative PCR
OSB 16	5′ TGA CCA AAC TAC TTA CAA CTC C 3′	Forward primer used to amplify *ACT1* for semiquantitative PCR
OSB 14	5′ TTA GAA ACA CTT GTG GTG AAC G 3′	Reverse primer used to amplify *ACT1* for semiquantitative PCR
OSB 381	5′ TAT TTC TTG TCC TAT CTT C 3′	Reverse primer used to amplify N-466 of *chl1*
OSB 382	5′ TCG AGA GAT CCA AAC AAT GGC 3′	Forward primer used to amplify C-648 of *chl1*
OSB 383	5′ TCG TGC AAT CAT GTT ATA CCG 3′	Forward primer used to amplify C-253 of *chl1*
OSB 384	5′ GTG AGG AAA ATA TTC TAT GAA GC 3′	Forward primer used to amplify C-170 of *chl1*

### Yeast strains.

The strains used in this study are listed in Table 2. For monitoring the level of Chl1, we incorporated the *MYC* tag at the C-terminal end of *CHL1* in isogenic wild-type, *Δhsc82*, and *Δhsp82* strains and in the temperature-sensitive *iG170Dhsp82* strain to generate NKY2, NKY39, NKY40, NKY41, and NKY45 strains. To determine the level of Chl1 in the presence of 17-AAG, we knocked out *PDR5* from the NKY2 strain to generate NKY43, so that maximum uptake of 17-AAG is ensured. For the sister chromatid cohesion assay, we used NKY4 as a parental strain. To visualize the expression of *PDS1* under our assay condition, we MYC tagged *PDS1* in NKY4 to generate NKY9. To perform the sister chromatid cohesion assay in the presence of 17-AAG, *PDR5* was knocked out in NKY9 to generate NKY61. Also, to perform the same in the absence of *CHL1*, *CHL1* was knocked out from NKY9 to generate NKY62. The parental strain used for chromosome loss assay is YMH58a. *CHL1* and *PDR5* knockouts were created in this background to generate NKY46 and NKY47, respectively. We have transformed truncated Chl1 (pLAchl1-N466), full-length Chl1, and empty vector individually to the NKY46 strain to create SBY1, SBY2, and SBY3 strains, respectively. To perform yeast two-hybrid analysis, the PJ69-4A strain was employed (37). We have transformed p*GBDUC1*/*HSP82* and p*GADC1*/*CHL1* into the PJ69-4A strain to generate NKY49. The transformants were selected in medium lacking uracil and leucine. In order to check for self-activation of bait and prey fusion products, NKY48, NKY50, and NKY51 were created. To map the domains of Chl1 that are responsible for interaction with Hsp90, we have transformed four truncated *chl1*-prey vectors, p*GADC1chl1-N466*, p*GADC1chl1-C648*, p*GADC1chl1-C253*, and p*GADC1chl1-C170*, to the p*GBDUC1HSP90*-containing strain to generate NKY56, NKY57, NKY58, and NKY59, respectively. For checking self-activation of the truncated *chl1*-prey vectors, NKY52, NKY53, NKY54, and NKY55 strains were generated.

**TABLE 2:** Yeast strains used in this study

Strain	Genotype	Source or reference
W303α	*MATα 15ade2-1 ura3-1,112 his3-11 trp1 leu2-3*	This study
NKY39	*MAT***a** *15ade2-1 ura3-1,112 his3-11 trp1 leu2-3 VIIL*::*ADE2 CHL1-13MYC*::*TRP*	This study
NKY40	*MAT***a** *15ade2-1 ura3-1,112 his3-11 trp1 leu2-3 VIIL*::*ADE2 HSC82*::*KAN*^r ^*CHL1-13MYC*::*TRP*	This study
NKY41	*MAT***a** *15ade2-1 ura3-1,112 his3-11 trp1 leu2-3 VIIL*::*ADE2 HSP82*::*KAN*^r ^*CHL1-13MYC*::*TRP*	This study
NKY43	*MATα 15ade2-1 ura3-1,112 his3-11 trp1 leu2-3 CHL1-13MYC*::*TRP Δpdr*::*loxP-leu2-loxP*	This study
NKY2	*MATα 15ade2-1 ura3-1,112 his3-11 trp1 leu2-3 CHL1-13MYC*::*TRP*	This study
NKY3	*MATα 15ade2-1 ura3-1,112 his3-11 trp1 leu2-3 chl1*::*TRP*	This study
SLY89	*Δhsc82*::*kanMX4Δhsp82*::*kanMX4/piHGpd-G170Dhsp82-HIS Δpdr*::*loxP-leu2-loxP* *trp1-289 leu2-3,112 his3-Δ200URA 3-52 ade2-101Δc lys2-801 am*	This study
NKY45	*Δhsc82*::*kanMX4Δhsp82*::*kanMX4/piHGpd-G170Dhsp82-HIS Δpdr*::*loxP-leu2-loxP trp1-289* *leu2-3,112 his3-Δ200URA 3-52 ade2-101Δc lys2-801 am CHL1-13MYC*::*TRP*	This study
*7D MAT***a**	*7D MAT***a** *SPC29-CFP*::*KAN mRFP-TETR URA3*::*TETO* *GFP-LACI*::*LEU2 YCPlac112GAL CEN LACO-TRP1*	Gift from Santanu K. Ghosh
NKY4	*7D MAT***a** *SPC29-CFP*::*KAN mRFP-TETR URA3*::*TETO GFP-LACI*::*LEU2 YCPlac112GAL*	This study
NKY9	*7D MAT***a** *SPC29-CFP*::*KAN mRFP-TETR URA3*::*TETO* *GFP-LACI*::*LEU2 YCPlac112GAL PDS1-13MYC*::*TRP*	This study
NKY61	*7D MAT***a** *SPC29-CFP*::*KAN mRFP-TETR URA3*::*TETO* *GFP-LACI*::*LEU2 YCPlac112GAL PDS1-13MYC*::*TRP pdr5*::*HIS3*	This study
NKY62	*7D MAT***a** *SPC29-CFP*::*KAN mRFP-TETR URA3*::*TETO* *GFP-LACI*::*LEU2 YCPlac112GAL chl1*::*HIS3 PDS1-13MYC*::*TRP*	This study
YMH58a	*MAT***a** *15ade2-1 ura3-1,112 his3-11 trp1-1 leu2-3 CFIII* (*CEN3.L.YMH58*) *URA3 SUP11*	Gift from Akash Gunjan
NKY46	*MAT***a** *15ade2-1 ura3-1,112 his3-11 trp1-1 leu2-3 CFIII* (*CEN3.L.YMH58*) *URA3 SUP11 chl1*::*TRP*	This study
NKY47	*MAT***a** *15ade2-1 ura3-1,112 his3-11 trp1-1 leu2-3 CFIII* (*CEN3.L.YMH58*) *URA3 SUP11 pdr5*::*TRP*	This study
SBY1	*MAT***a** *15ade2-1 ura3-1,112 his3-11 trp1-1 leu2-3* *CFIII* (*CEN3.L.YMH58*) *URA3 SUP11 chl1*::*TRP* pLA*chl1-N-466*	This study
SBY2	*MAT***a** *15ade2-1 ura3-1,112 his3-11 trp1-1 leu2-3* *CFIII* (*CEN3.L.YMH58*) *URA3 SUP11 chl1*::*TRP* pLA*CHL1*	This study
SBY3	*MAT***a** *15ade2-1 ura3-1,112 his3-11 trp1-1 leu2-3* *CFIII* (*CEN3.L.YMH58*) *URA3 SUP11 chl1*::*TRP* pLA	This study
PJ69-4A	*MAT***a** *trpl-901 leu2-3,112 ura3-52 his3-200 ga14Δ* *ga180Δ LYS2*::* GAL1-HIS3 GAL2-ADE2 met2*::*GAL7-lacZ*	37
NKY48	*MAT***a** *trpl-901 leu2-3,112 ura3-52 his3-200 ga14Δ ga180Δ LYS2*::* GAL1-HIS3 GAL2-ADE2* *met2*::*GAL7-lacZ* p*GBDUC1* p*GADC1*	This study
NKY49	*MAT***a** *trpl-901 leu2-3,112 ura3-52 his3-200 ga14Δ ga180Δ LYS2*::* GAL1-HIS3 GAL2-ADE2* *met2*::*GAL7-lacZ* p*GBDUC1/HSP82* p*GADC1/CHL1*	This study
NKY50	*MAT***a** *trpl-901 leu2-3,112 ura3-52 his3-200 ga14Δ ga180Δ LYS2*::* GAL1-HIS3 GAL2-ADE2* *met2*::*GAL7-lacZ* p*GBDUC1/HSP82* p*GADC1*	This study
NKY51	*MAT***a** *trpl-901 leu2-3,112 ura3-52 his3-200 ga14Δ ga180Δ LYS2*::*GAL1-HIS3 GAL2-ADE2* *met2*::*GAL7-lacZ* p*GBDUC1* p*GADC1/CHL1*	This study
NKY52	*MAT***a** *trpl-901 leu2-3,112 ura3-52 his3-200 ga14Δ ga180Δ LYS2*::* GAL1-HIS3 GAL2-ADE2* *met2*::*GAL7-lacZ* p*GBDUC1* p*GADC1/chl1-N466*	This study
NKY53	*MAT***a** *trpl-901 leu2-3,112 ura3-52 his3-200 ga14Δ ga180Δ LYS2*::* GAL1-HIS3 GAL2-ADE2* *met2*::*GAL7-lacZ* p*GBDUC1* p*GADC1/chl1-C648*	This study
NKY54	*MAT***a** *trpl-901 leu2-3,112 ura3-52 his3-200 ga14Δ ga180Δ LYS2*::* GAL1-HIS3 GAL2-ADE2* *met2*::*GAL7-lacZ* p*GBDUC1* p*GADC1/chl1-C253*	This study
NKY55	*MAT***a** *trpl-901 leu2-3,112 ura3-52 his3-200 ga14Δ ga180Δ LYS2*::* GAL1-HIS3 GAL2-ADE2* *met2*::*GAL7-lacZ* p*GBDUC1* p*GADC1/chl1-C170*	This study
NKY56	*MAT***a** *trpl-901 leu2-3,112 ura3-52 his3-200 ga14Δ ga180Δ LYS2*::* GAL1-HIS3 GAL2-ADE2* *met2*::*GAL7-lacZ* p*GBDUC1/HSP82* p*GADC1/chl1-N466*	This study
NKY57	*MAT***a** *trpl-901 leu2-3,112 ura3-52 his3-200 ga14Δ ga180Δ LYS2*::* GAL1-HIS3 GAL2-ADE2* *met2*::*GAL7-lacZ* p*GBDUC1/HSP82* p*GADC1/chl1-C648*	This study
NKY58	*MAT***a** *trpl-901 leu2-3,112 ura3-52 his3-200 ga14Δ ga180Δ LYS2*::* GAL1-HIS3 GAL2-ADE2* *met2*::*GAL7-lacZ* p*GBDUC1/HSP82* p*GADC1/chl1-C253*	This study
NKY59	*MAT***a** *trpl-901 leu2-3,112 ura3-52 his3-200 ga14Δ ga180Δ LYS2*::* GAL1-HIS3 GAL2-ADE2* *met2*::*GAL7-lacZ* p*GBDUC1/HSP82* p*GADC1/chl1-C170*	This study

### Treatment with inhibitors.

For treatment with 17-AAG, the cells were grown until they reached an optical density at 600 nm (OD_600_) of 0.3 at 30°C. Next, we added 17-AAG at the working concentration of 40 µM and cells were allowed to grow overnight. In the case of experiments which required mid-log-phase cells, a secondary inoculum was given the next day in the presence of 17-AAG, and the cells were grown until the required OD_600_ was reached. For treatment with MG132, the NKY45 strain was grown at 37°C overnight in the presence of MG132 at the working concentration of 50 µM.

### Western blotting.

For protein extraction, exponentially growing cells of the strains NKY2 and NKY43 were taken. To achieve the functional loss of Hsp82 in NKY45 strains, one batch of cells was grown at 37°C overnight, and the other batch of cells was allowed to grow at 25°C. Equal amounts of cells were finally harvested, and protein was isolated from them by the trichloroacetic acid (TCA) method and subsequently followed for Western blotting (39). The antibodies used were mouse anti-Act1 antibody (Abcam) and mouse anti-Hsp82 antibody (Calbiochem) at 1:5,000 dilutions. Rabbit anti-Myc antibody (Abcam) was used at 1:8,000 dilutions. For secondary antibodies, horseradish peroxide-conjugated anti-rabbit antibody (Promega) and anti-mouse antibody (Santa Cruz Biotechnology Inc., CA, USA) were used at 1:10,000 dilutions. The Western blots were developed using a chemiluminescent detection system (Pierce). The bands on the blots were quantified using ImageJ software, and the relative densities thus obtained were plotted using GraphPad Prism 6 software. The mean values from three independent experiments were plotted (± standard deviation [SD]).

### RNA isolation and semiquantitative PCR.

RNA isolation and cDNA preparation from two batches of NKY1 strains were performed by growing the strain at 25°C and 37°C overnight, respectively. The cells corresponding to an OD_600_ of 10 were harvested, and total RNA was then isolated by using the acid phenol method as described previously (39). The cDNA was synthesized in the same way as depicted in the above reference and then subjected to PCR amplification (27 cycles) with gene-specific primers to score for *CHL1* transcription by amplifying 262 bp at the 3′ end of the transcript. As a normalization control, the *ACT1* transcript was amplified, corresponding to 307 bp.

### Co-IP assay.

Wild-type and *Δchl1* cells were grown until mid-logarithmic phase in 10 ml medium. We performed the coimmunoprecipitation (Co-IP) assay using the protocol described previously (6). The anti-Hsp82 antibody was used to immunoprecipitate Hsp82. Western blotting was then performed with the immunoprecipitate obtained along with the supernatant and total cellular protein of the cell serving as input. The membrane was probed with anti-Myc antibody to witness the physical interaction between Hsp90 and Chl1. The control antibody used for Co-IP was rabbit IgG.

### Yeast two-hybrid analysis.

For yeast two-hybrid analysis, *HIS3* reporter gene expression was monitored as the readout of protein-protein interactions. The cells were grown to an OD_600_ of 0.5 and then subjected to serial dilutions. The diluted cells were then spotted simultaneously on two plates: one lacking uracil and leucine and the other lacking uracil, leucine, and histidine. Growth on these plates was scored after 5 days of incubation at 30°C. The self-activation was scored for baits in PJ69-4A, and the lack of growth ensured that the bait fusions did not lead to self-activation.

### Sister chromatid cohesion assay.

The percentage of sister chromatid cohesion was determined by indirect immunofluorescence. The NKY61 and NKY62 strains were grown until reaching an OD_600_ of 0.2 in an appropriate selective medium. Nocodazole was then added at the final concentration of 15 µg/ml, and the cells were allowed to grow for 1.5 h to 2 h to achieve a high number of budding cells, which was confirmed by observing them under the microscope. After the complete arrest of a maximum number of cells, 10^7^ to 10^8^ cells from each strain were harvested by centrifugation. The spheroplasts were fixed on a slide with poly-l-lysine (Sigma) and incubated with anti-RFP as primary antibody at a dilution of 1:200 (Allied Scientific) overnight at 4°C. This step was followed by washing the slide five times with buffer W.T. (1% nonfat dry milk, 200 mM NaCl, 50 mM HEPES-KOH, pH 7.5, 1 mM NaN_3_, 0.1% Tween 20). Incubation with anti-goat–Alexa Fluor 594 (Invitrogen catalog no. A-11032) at 1:200 dilutions was carried out for 90 min in the dark. The samples were then washed with buffer W.T. five times. Slides were covered with a mounting solution and observed under a confocal fluorescence microscope (Zeiss LSM 510 Meta). The mean values (±SDs) from three independent experiments were plotted using GraphPad Prism 6 software.

### Chromosome loss assay.

Strains NKY46 and NKY47 were grown overnight in synthetic complete (SC)-Ura medium. Similarly, SBY1, SBY2, and SBY3 were grown in the SC-Ura-Leu medium. The next day, the secondary inoculum was given such that the starting OD_600_ was 0.1. The cells were allowed to grow until reaching an OD_600_ of 0.3 to 0.4. Cells to the number of 10^4^ were then spread from each culture on the yeast extract-peptone-dextrose (YPD) plates. After the colonies were obtained, the plates were kept at 4°C for 5 to 6 days for color development. For determining first-division chromosome loss, only the sectored colonies showing at least 50% but not 100% red color were taken into account. We calculated the number of at least 50% red colonies in wild-type cells as well as the *chl1* null strain or the 17-AAG treatment condition. Fold change in the chromosome loss frequencies was plotted. The mean values (±SDs) from three independent experiments were plotted using GraphPad Prism 6 software.
